# Perceived Symptoms of Depression, Anxiety and Stress amongst Staff in a Malaysian Public University: A Workers Survey

**DOI:** 10.3390/ijerph182211874

**Published:** 2021-11-12

**Authors:** Mohd Rizal Abdul Manaf, Muhammad Al-Amin Shaharuddin, Azmawati Mohammed Nawi, Noorlaili Mohd Tauhid, Hanita Othman, Mohd Rizam Abdul Rahman, Hanizah Mohd Yusoff, Nazarudin Safian, Pei Yuen Ng, Zahara Abdul Manaf, Nor Ba’yah Abdul Kadir, Kevina Yanasegaran, Siti Munirah Abdul Basir, Sowmya Ramakrishnappa, Mohd Izhar Ariff, Kurubaran Ganasegeran

**Affiliations:** 1 Department of Community Health, Faculty of Medicine, Universiti Kebangsaan Malaysia, Kuala Lumpur 56000, Malaysia; p108697@siswa.ukm.edu.my (M.A.-A.S.); azmawati@ppukm.ukm.edu.my (A.M.N.); rizam@ppukm.ukm.edu.my (M.R.A.R.); drhanie@ppukm.ukm.edu.my (H.M.Y.); nazarudin@ppukm.ukm.edu.my (N.S.); drsonygowda2007@gmail.com (S.R.); 2 Department of Family Medicine, Faculty of Medicine, Universiti Kebangsaan Malaysia, Kuala Lumpur 56000, Malaysia; laili@ppukm.ukm.edu.my; 3 Department of Pathology, Faculty of Medicine, Universiti Kebangsaan Malaysia, Kuala Lumpur 56000, Malaysia; drhanita@ppukm.ukm.edu.my; 4 Drug and Herbal Research Centre, Faculty of Pharmacy, Universiti Kebangsaan Malaysia, Kuala Lumpur 50300, Malaysia; pyng@ukm.edu.my (P.Y.N.); ykevina@yahoo.com (K.Y.); 5 Dietetic Program, Faculty of Health Sciences, Universiti Kebangsaan Malaysia, Kuala Lumpur 50300, Malaysia; zaharamanaf@ukm.edu.my (Z.A.M.); sitimunirah.abdulbasir@gmail.com (S.M.A.B.); 6 Centre for Research in Psychology and Human Well-Being, Faculty of Social Sciences and Humanities, Universiti Kebangsaan Malaysia, Bangi 43600, Malaysia; aknbayah@ukm.edu.my; 7 Department of Medicine, Faculty of Medicine, Universiti Kebangsaan Malaysia, Kuala Lumpur 56000, Malaysia; izhar.ariff@ppukm.ukm.edu.my; 8 Clinical Research Center, Seberang Jaya Hospital, Ministry of Health Malaysia, Penang 13700, Malaysia

**Keywords:** depression, anxiety, stress, workers, university, Malaysia

## Abstract

Mental health conditions are a major part of workers’ health that predisposes to poor self-motivation for sustaining productivity. This study was aimed to determine the prevalence of depression, anxiety, and stress among staff in a Malaysian public university and its associated factors. A cross-sectional study was conducted among 459 staff from the Universiti Kebangsaan Malaysia (UKM) between April and June 2019. A questionnaire that consisted of items on socio-demographic and socioeconomic characteristics, employment description, lifestyle risk behaviors, personal medical history, and symptoms of depression, anxiety, and stress was administered to participants. Descriptive and inferential statistics were conducted using SPSS version 22.0. The prevalence of perceived symptoms of depression, anxiety, and stress among the respondents was 28.7%, 50.1%, and 14.8%, respectively. Over one-quarter (26.5%) of the participants presented symptoms of two or more mental disorders. Women, those aged less than 40 years old, and non-academic professionals were more likely to exhibit depressive symptoms, while those with medical conditions that required hospitalizations sustained anxiety symptoms. Perceived stress was more likely to be prevalent among staff with secondary education or less and smokers. Proactive support for staff needs to be offered in sustaining their emotional well-being.

## 1. Introduction

Approximately 450 million people globally were affected by mental ailments [1], and it remains one of the leading causes for the overall disease burden [2]. The pooling of multi-country epidemiological data revealed that at least one in five adults experienced a common mental disorder within the past twelve months while projecting that approximately 29.2% of the global adult population could have experienced certain psychological illnesses [3]. Across the South Asian countries, the prevalence of depression was reported to be 26.4%, while anxiety accounted for 25.8% of the total population [4]. Malaysia recorded a three-fold increase in the prevalence of mental health repercussions among adults aged 16 years and above, from 10.7% in 1996 to 29.2% in 2015 [5]. Depression, anxiety, and stress were the common mental health conditions reported. These conditions are considered vital indicators for mental health that, if left untreated, would pose a negative effect on individuals [6,7].

Depression is one of the main causes of disability worldwide and a major contributor to the burden of suicide and ischemic heart disease [8]. Experts forecast that by 2030, depression is likely to be the third leading cause of disease burden in low-income countries and the second highest cause of disease burden in middle-income countries [9]. Depressive disorders and several other mental health conditions are among the top 10 causes of disability due to health-related conditions in low and middle-income countries, representing 19.1% of all disability related to health conditions [10]. Nationally, 2.3% of Malaysian adults were affected by depression [11]. Meanwhile, the prevalence of anxiety disorders across the world varied from 2.5% to 7% by country globally, as almost 284 million people experienced anxiety disorders as of 2017 [12]. Stress and anxiety are both emotional responses. The difference between these two mental health conditions is that stress is triggered by external stimuli, whereas anxiety persists without any stressors [13]. Mental disorders are on the rise in every country in the world, and it will cost the global economy an estimated $16 trillion by 2030 [14]. The economic cost is primarily due to early onset of mental illness and lost productivity, with an estimated 12 billion working days lost due to mental illness every year. Mental health conditions in the workplace are estimated to cost the Malaysian economy RM14.46 billion in 2018 with RM344.82 million or 1% of the health budget spent on mental health treatment [15].

Studies on mental health in higher education facilities tend to focus on the students as compared to the employees. However, concerns about mental health in the research community have been growing in recent years [16]. Among the mental health conditions, stress and burnout were the most commonly reported conditions. Survey data showed that the majority of university staff found their job to be stressful with levels of burnout apparently higher among university staff than the general working populations. Such mental health conditions in tertiary institutions were comparable to “high-risk” groups such as healthcare workers [17]. In Malaysia, it was found that the prevalence of stress among educators from higher learning institutions (colleges and universities) ranged from 5.5% to 25.9% [18]. Several factors can contribute to the high prevalence of stress among individuals from this occupation group, including a hypercompetitive academic environment in which employees often need to direct all their attention and energy onto work with the absence of formal or informal support from the institutions [19]. Mental health conditions, such as depression and anxiety, may result in absenteeism, which in turn will cause a reduction in job performance and work productivity [9]. Furthermore, the negative effects of poor mental health status may not be limited only to the quality of life of the workers but also the achievement of the institution [20]. Prevention and early intervention are important so that appropriate and prompt management can be provided to the patients. Therefore, it is essential to identify those workers who are at a higher risk of developing mental health problems.

Despite the increasing prevalence of mental health conditions in Malaysia, data on specific occupational groups, such as among university staff, are limited. Studies on the mental health conditions of university workers have received less attention to date as compared to the bulk of literature that explored such study objectives amongst university students as the sampling frame. To reflect a turning point, it is essential to study such conditions from the provider perspectives (mentors, educators, and administrators) as compared to the end-users (students) in an academic institution to form a paradigm shift of understanding a predictor-led needs–gap framework of mental health repercussions within the university delivery setting in the quest to reduce the risk of overwhelmed faculties while nurturing future leaders. This study was aimed to determine the prevalence of depression, anxiety, and stress among staff in a Malaysian public university and its associated factors. The current study postulated that female workers, non-academic professionals, younger aged workers, those with medical conditions that required hospitalizations, those with secondary education or less, and smokers would be subjected to greater odds of perceived symptoms of depression, anxiety, or stress.

## 2. Materials and Methods

### 2.1. Study Design, Setting and Participants

This cross-sectional study was conducted from April to June 2019 among staff at the Universiti Kebangsaan Malaysia (UKM), Bangi, Selangor, Malaysia.

### 2.2. Sample Size Calculation

Based on a sample size calculation for prevalence studies [21], a minimum sample size of 383 staff was calculated to represent a cross-section of the population and to allow the study to determine the prevalence of depression, anxiety, and stress among the staff. An additional 30% was included in the calculated sample to compensate for missing data and non-response, for a final sample size of 497 staff. Four hundred and ninety-seven staff, both academics and non-academics, were randomly invited to participate in the study. Random selection was conducted using a free computer aided software (Research Randomizer) [22]. The sampling frame included the entire university’s staff population, with their employment identity number provided by the Department of Registrar, Universiti Kebangsaan Malaysia. The single generated set of 497 random samples was identified, and subsequently, an invitation was sent out to the employees through their official email registered in the personal profile. The study was conducted at the hall or foyer of selected faculties and departments in the university.

### 2.3. Study Inclusion and Exclusion Criteria

Permanent and contract staff aged between 18 and 60 years old were included in the study. Pregnant or breast-feeding staff and those who were on maternity leave or sabbatical were excluded.

### 2.4. Study Instrument

Respondents were invited to complete a self-administered questionnaire that consisted of items on socio-demographic characteristics (gender, age, and marital status), socioeconomic characteristics (household income and education level), employment description (occupation level and duration of service years) risky lifestyle behaviors (smoking status and physical inactivity), personal medical history (presence of medical conditions that required hospitalizations such as stroke, myocardial infarction, chronic kidney disease, tuberculosis, acute asthma), and perceived symptoms of depression, anxiety, and stress.

Participants’ household income was classified into three categories—Top 20% (T20: ≥2610 USD), Middle 40% (M40: 1155-<2610 USD), and Bottom 40% (B40: <1155 USD)—based on latest household income classification in Malaysia [23]. Participants’ occupation levels were divided into academics, non-academic professionals, and non-academic support staff. Smokers were defined as those who have smoked at least 100 cigarettes during their lifetime [24]. The item was assessed using a dichotomized response (yes/no).

Physical activity (PA) was assessed using the validated Malay version of the Global Physical Activity Questionnaire (GPAQ-M) [25]. The GPAQ-M comprises of 16 questions that asked participants about the intensity, frequency, and duration of PA across 3 major domains, namely PA at work, PA during travel or transport, and PA during recreation or leisure time, in addition, to an extra question that collected data on sedentary behavior and time in minutes/day. Metabolic equivalent task (MET) is defined as ‘the ratio of a person’s working metabolic rate relative to their resting metabolic rate’ [26]. A metabolic equivalent task (MET) value of 4 was designated as moderate intensity PA, while a value of 8 was assigned as vigorous intensity PA. These values of MET were subsequently multiplied by the number of days per week of PA and the duration on a typical day for each PA domain to tabulate the total PA (MET-min/week). The MET-minutes/week spent on each domain was subsequently computed to yield the overall PA level. The PA level was classified into two categories, active PA level and inactive PA level, based on recommendation by the WHO [27]. Active PA level was defined as participants who achieved a minimum of at least 600 MET-minutes/week. Participants who did not meet the criteria were classified as having an inactive PA level. Personal medical history was assessed based on each respondent’s self-reported medical conditions as diagnosed by a doctor or under current use of medications.

Perceived symptoms of depression, anxiety, and stress were assessed using the validated Malay version of the 21-items Depression, Anxiety and Stress Scale (DASS-21) [28]. The scale consists of 3 sub-domains of seven items each. Respondents will rate the extent to which each statement applies to them during the past week on a 4-point Likert scale ranging from 0 (did not apply to me at all) to 3 (applied to me very much). The depression scale assesses dysphoria, hopelessness, devaluation of life, self-deprecation, lack of interest/involvement, anhedonia, and inertia. The anxiety scale assesses autonomic arousal, skeletal muscle effects, situational anxiety, and subjective experience of anxious affect. The stress scale is sensitive to levels of chronic nonspecific arousal. It assesses difficulty relaxing, nervous arousal, and being easily upset/agitated, irritable/over-reactive, and impatient. Since the DASS 21 is the short-form version of the original DASS (42 items), the final score for each subscale is multiplied by two and evaluated to its severity rating index. Scores for depression, anxiety, and stress are calculated by summing the scores for the relevant items [29]. The results are interpreted as follows: Depression (>27 = extremely severe depression; 27–21 = severe depression; 20–14 = moderate depression; 13–10 = mild depression and 9–0 = no depression/normal), Anxiety (>19 = extremely severe anxiety; 19–15 = severe anxiety; 14–10 = moderate anxiety; 9–8 = mild anxiety and 7–0 = no anxiety/normal), and Stress (>33 extremely severe stress; 32–26 = severe stress; 25–19 = moderate stress; 18–15 = mild stress; and 14–0 = no stress/normal).

### 2.5. Statistical Analyses

Analysis was conducted using IBM SPSS Statistics version 22.0 [30]. Descriptive statistics were conducted for all variables in the study. Pearson chi-square test and binary logistic regressions were used to assess the associations between perceived symptoms of depression, anxiety, and stress with sample characteristics. Crude odds ratios (cOR) and effect estimates were reported.

Multiple logistic regression analysis using ‘Backward’, ‘Forward’, and ‘Enter’ regression techniques was employed to determine the factors associated with perceived symptoms of depression, anxiety, and stress in this sample. Adjusted odds ratios (aOR) and effect estimates were reported. Multicollinearity between independent variables were checked. Statistical significance was set at *p* < 0.05.

## 3. Results

### 3.1. Sample Characteristics

Table 1 shows sample characteristics. A total of 459 staff (32.7% men and 67.3% women) participated in this study. The mean (SD) age of the respondents was 43.21 (7.57) years and the age ranged between 29 and 60 years old. More than half of the respondents were aged 40 years or more, 278 (60.6%). The majority were married, 395 (86.1%), and 352 were tertiary educated (76.7%). Nearly half (198, 47.5%) of the participants were classified within the M40 household income group. Two hundred and sixty-one (56.9%) of the participants were non-academic support staff, while the majority, 234 (51.0%), had been in service for more than 15 years.

Seventy-three participants (15.9%) were physically inactive, and sixty (17.4%) of them were smokers. Only 16 participants (3.5%) from the total sample suffered medical conditions that required hospitalizations.

### 3.2. Prevalence of Depression, Anxiety and Stress

**Figure 1:**
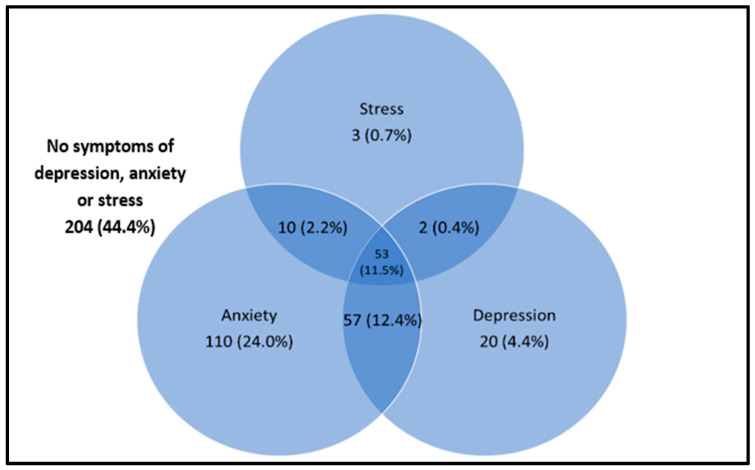
Coexistence of symptoms of depression, anxiety, and stress (*n* = 459).

Cronbach’s alpha values for anxiety, depression, and stress subscales for the current sample were 0.783, 0.847, and 0.825 respectively, showing acceptable to good internal consistencies of the DASS-21 instrument. A total of 255 (55.6%) participants perceived to have symptoms of at least one mental health condition. Of the total sample, 230 (50.1%) had symptoms of anxiety, 132 (28.7%) had symptoms of depression, and 68 (14.8%) had symptoms of stress (Table 2). The most common isolated mental health condition among the participants was anxiety (110, 24.0%), which was followed by depression (20, 4.4%) and stress (3, 0.7%). Sixty-nine (15.0%) of the participants presented symptoms of two mental health conditions, the most common being the combinations of anxiety and depression, which accounted for 57 (12.4%) of the participants. Fifty-three (11.5%) of the participants experienced symptoms of depression, anxiety, and stress simultaneously (Figure 1).

### 3.3. Factors Associated with Depression, Anxiety and Stress at the Bivariate Level

Depression was higher among women (cOR 1.76; 95% CI 1.12, 2.79), those aged less than 40 years old (cOR 1.62; 95% CI 1.07, 2.44), those within the B40 household income group (cOR 2.44; 95% CI 1.31, 4.55), non-academic professionals (cOR 2.15; 95% CI 1.21, 3.81), and those being in service for 15 years or less (cOR 1.55; 95% CI 1.03, 2.33). Meanwhile, anxiety was found to be higher among those who suffered medical conditions that required hospitalizations (cOR 2.39; 95% CI 1.26, 4.52). These associations were statistically significant (Table 3).

### 3.4. Factors Associated with Depression, Anxiety and Stress by Multiple Logistic Regression

All variables with *p*-values less than 0.20 (*p* < 0.20) at the univariate level were included in the multivariate analysis. For perceived symptoms of depression, the multivariable analysis retained three factors in the final model: being women (aOR 1.62; 95% CI 1.01, 2.65), those aged less than 40 years old (aOR 1.58; 95% CI 1.02, 2.45), and non-academic professionals (aOR 2.04; 95% CI 1.12, 3.72). With regard to perceived symptoms of anxiety, the multivariable model confirmed that those who suffered medical conditions that required hospitalizations had almost twice the odds of being anxious as compared to those without such conditions (aOR 2.38; 95% CI 1.24, 4.50). For perceived symptoms of stress, the multivariable model retained two factors in the final model: those with secondary education or less (aOR 2.20; 95% CI 0.90, 5.42) and smokers (aOR 2.56; 95% CI 0.88, 7.40). There was no multicollinearity between independent variables.

## 4. Discussion

### 4.1. Core Summary Findings

This study aimed to determine the prevalence of self-reported depression, anxiety, and stress symptoms among staff in a Malaysian public university and its associated factors. There is a moderate prevalence of perceived depression, anxiety, and stress symptoms in this sample with more than half of the study sample reported to exhibit at least one mental health condition. Women, those aged less than 40 years old, and non-academic professionals were more likely to perceive symptoms of depression, while those with medical conditions that required hospitalizations perceived symptoms of anxiety. Perceived stress was more likely to be prevalent among staff who were smokers and those with secondary education or less.

### 4.2. Comparisons with Existing Literature

Consistencies on the prevalence rates of self-reported mental health conditions in the literature have shown mixed variations. A study among faculty members from the USA reported that the prevalence of depression and anxiety was 28.3% and 38.6%, respectively [31], which is lower than that reported in the current study. However, that same study reported higher prevalence rates of stress [31] when compared to the findings of the current study. In contrast, university staff from Southwest Ethiopia showed lower depression (22.9%), anxiety (19.2%), and stress (28.2%) rates [32] when compared to the findings of this study. A study done in 17 Australian universities found that 43% of academic staff and 37% of non-academic staff experienced occupational stress [33], while another study from China reported the prevalence of depressive symptoms to be 58.9% among university teachers [34]. From the local setting, mental health surveys conducted among public university staff at different institutions found that the prevalence of depression, anxiety, and stress ranged between 21.7% and 70.5% [35,36].

Compared to other occupational groups, the prevalence of depression, anxiety, and stress among university staff in this study was found to be lower than the prevalence rates reported among midwives [37] and waiters of upscale restaurants [38] but higher than among hospital workers [39], nurses [40], magistrates [41], and emergency medical service professionals [42]. The difference in the prevalence of mental health conditions in different occupational groups may be contributed by the difference in job characteristics such as job demand, job control, as well as job strain [43]. Variations in the prevalence rates of depression, anxiety, and stress could be attributed to the utilization of different study tools across different studies, which were adopted for a variety of study populations or occupational settings. Those investigations that adopted an arbitrary criterion for cut-off points or dichotomizing sub-domain scores may pose unstable estimates on the prevalence rates of the outcomes being studied. Such circumstances may pose possible threats to validity when adapted cross-culturally in different occupation settings, confounded with the organizational work climate to measure depression, anxiety, and stress.

A small proportion of the current study participants presented with symptoms of two psychological disorders, while a little over one-tenth reported to have symptoms of all the psychological disorders. This association has been previously described both in the general population [44] as well as among university staff [35]. A prospective study on the temporal dynamics and longitudinal co-occurrence of depression and different anxiety syndromes in youth suggests that there is a bidirectional, systematic pattern between the development of depressive and anxious syndromes in young adults [45]. This study found that women had higher odds of having symptoms of depression as compared to men. This finding was consistent with other studies from different countries [32,46,47,48]. Similarly, anxiety and stress were more likely to have occurred in women, but this finding was not sufficiently powered to be retained in the final regression models. The link between depression and women can be explained from a socioeconomic as well as from a biological point of view. The difference in socioeconomic characteristics such as education and income may have resulted in higher rates of depression among women [49]. Women and men react differently to stressors and may be more vulnerable to develop depression and anxiety-related disorders [50]. Biological factors, such as hormonal imbalances, may also play a role, which could have resulted in higher odds of depression among women. Although established evidence from a longitudinal cohort study found that occupational settings and job-related factors have accelerated the risk of postpartum depression [51], and it is worthwhile to note that the current study was not powered enough to establish such relationships in view of the study design that was cross-sectional in nature. Cross-sectional studies act as a “snapshot” for “prevalence” to be determined and could only explore the psychological repercussion of workers within that point of time; hence, investigators in the current study had to be careful in avoiding erroneous interpretation of synthesized results. Owing to physiological psychological interference, such as antenatal depression or anxiety during pregnancy that has the capacity to confound the current study findings, which should principally be determined by risk factors from an occupational perspective in an academic setting, pregnant workers were excluded from recruitment in this study.

Younger aged employees were at higher odds of having depressive symptoms as compared to older age groups. Previous studies regarding the association of age and symptoms of depression produced mixed results. While some studies found a negative relation between age and depression [52], studies from developed countries consistently found that the odds of depression decreased with age. In contrast, investigations from developing countries generally did not establish any causal associations between depression and age [53]. Studies found a linear interaction between depression and age, which was most commonly seen amongst those with impaired health [54] and those with lower education in older aged groups [55]. It could be postulated that those in older age groups tend to have higher income with longer service duration, thus exhibiting lower odds of psychological conditions in this study, which most likely may be due to financial and job stability among those older age groups.

Blue-collar workers were more likely to suffer from mental health conditions than white-collar workers [56]. However, a study from the United Kingdom found that groups with higher prevalence of common mental disorders were clerical and secretarial staff, sales, and personal and protective services workers; in contrast, crafts and related, ‘other’ professional occupations, and plant and machine operation workers had a lower prevalence of common mental illness [57]. The findings of this study were in line with the former postulation that found non-academic professionals to have higher odds of developing symptoms of depression as compared to non-academic support staff. Such a phenomenon could be due to different job characteristics within the job scope, as such psychological demands of a job were found to be consistently related to depressive disorders [58].

In this study, respondents with medical conditions that were hospitalized had higher odds of being anxious as compared to those without such conditions. This finding was consistent with a previous study that found anxiety to be prevalent in chronic disease patients as compared to the general population [59]. The complex mechanism to cope with medical illness requires self-determination to overcome the emotional shock of the diagnoses. When coping strategies collapse over time due to low psychological, emotional, and social support or being influenced by external stressors, these situations exacerbate anxious states among individuals afflicted with disease [54].

The university health center offers a wide range of medical services to the workers that includes regular clinic sessions by healthcare professionals. Health promotion activities are also organized to increase workers’ awareness regarding the importance of mental health. Apart from the substantial prevalence of symptoms of depression, anxiety, and stress found in the current study sample, a number of factors associated with these symptoms, including age, gender, occupation categories, the presence of medical conditions that required hospitalization, education level, as well as smoking status were identified. Identifying these risk factors can be helpful to formulate strategies for the early identification of mental health conditions, and focused psychological or behavioral interventions being instituted in a timely manner would promote better mental health well-being among the staff.

### 4.3. Study Limitations and Recommendations

The limitations of this study need to be acknowledged. Firstly, of the study tools used to measure the prevalence of depression, anxiety, and stress, the DASS-21 questionnaire is a suitable tool to screen for depressive, anxious, and stress disorders, and it may be useful to identify individuals who are at risk of being affected by these conditions. However, additional tools should be used to establish a formal diagnosis. Secondly, due to the study design employed in this investigation, the establishment of associations between variables is allowed, but the establishment of causality is not. Although there were some variables retained in the multivariate regression model (gender associated with depression, education level, and smoking status associated with stress), these variables showed non-statistical significance. It is worthy to note that although statistical significance did not prevail by chance, these variables have somewhat proved an effect on the main outcome. It may be due to the sample size of this study, which was not sufficiently powered to establish statistical significance; as such, future studies may warrant a larger sample size. The generalizability of the study findings may be limited due to the non-representative demographics of the study population with a comparatively small sample size from a single institution. However, the public academia setting is the same for all universities in the country under the custodian of the Ministry of Higher Education of Malaysia (MOHE); hence, the findings could be extrapolated within the same population frame. Lastly, as women presented the most severe forms of depression, anxiety, and stress as reflected in the current study, and since sex was a factor that showed significant difference between certain groups within the psychological measures, it would be interesting for future studies to explore some sex differences in terms of interaction effects with other sociodemographic variables. A possible consequence for such an observed finding could be due to the number of women respondents being almost double that of men; as such, some effects of associations would be more likely to be associated with women. Succinctly, the current study results do not warrant an interaction analysis, as there was no multi-collinearity between independent variables being detected. However, as cross-sectional studies are always “hypothesis generating” in nature rather than “hypothesis testing,” it is recommended for future studies to consider such interactions with a sense of coherence (SOC) analytics through construct validity applications based on theoretical subcomponents for a demographic–psychological interaction framework.

## 5. Conclusions

The prevalence of having at least one mental health condition in this sample was 55.6%. For specific mental health condition, 28.7% had symptoms of depression, 50.1% were anxious, and 14.8% had symptoms of stress. Women, those aged less than 40 years old, and non-academic professionals were at higher odds of being depressed, while those with medical conditions that required hospitalizations were likely to be more anxious. Smokers and those with a secondary education or less were more likely to be stressed. The findings of this study indicate that proactive support needs to be offered to university staff in order to help sustain their emotional wellbeing.

## Figures and Tables

**Table 1:** Sample characteristics (*n* = 459).

Characteristics		*n* (%)
Gender	Men	150 (32.7)
	Women	309 (67.3)
Age (years)	Mean (SD)	43.21 (7.57)
Age groups (years)	<40	181 (39.4)
	≥40	278 (60.6)
Marital status	Single	64 (13.9)
	Married	395 (86.1)
Household income classification (*n* = 417)	B40	120 (28.8)
	M40	198 (47.5)
	T20	99 (23.7)
Education level	Secondary or less	107 (23.3)
	Tertiary	352 (76.7)
Occupation categories	Academic	104 (22.7)
	Non-academic professional	94 (20.5)
	Non-academic support	261 (56.9)
Duration of service years	≤15	225 (49.0)
	>15	234 (51.0)
Smoking status (*n* = 344)	No	284 (82.6)
	Yes	60 (17.4)
Physical activity level	Active PA	386 (84.1)
	Inactive PA	73 (15.9)
Medical conditions that required hospitalizations	No	443 (96.5)
	Yes	16 (3.5)

**Table 2:** Total scores from the DASS-21 questionnaire and by gender (*n* = 459).

Domains	Symptom Severity	Total *n* (%)	Men *n* (%)	Women *n* (%)	*p*-Value
Depression	No depression	327 (71.2)	118 (78.7)	209 (67.6)	0.027
	Mild	69 (15.0)	14 (9.3)	55 (17.8)	
	Moderate	54 (11.8)	18 (12.0)	36 (11.7)	
	Severe	8 (1.7)	0 (0)	8 (2.6)	
	Extremely severe	1 (0.2)	0 (0)	1 (0.3)	
	Mean (SD)	6.42 (5.49)	5.97 (4.95)	6.64 (5.73)	0.222
Anxiety	No anxiety	229 (49.9)	78 (52.0)	151 (48.9)	0.805
	Mild	59 (12.9)	19 (12.7)	40 (12.9)	
	Moderate	136 (29.6)	44 (29.3)	92 (29.8)	
	Severe	35 (7.6)	9 (6.0)	26 (5.7)	
	Extremely severe	0 (0)	0 (0)	0 (0)	
	Mean (SD)	7.67 (5.23)	7.45 (4.90)	7.78 (5.40)	0.531
Stress	No stress	391 (85.2)	133 (88.7)	258 (83.5)	0.479
	Mild	45 (9.8)	11 (7.3)	34 (11.0)	
	Moderate	22 (4.8)	6 (4.0)	16 (5.2)	
	Severe	1 (0.2)	0 (0)	1 (0.3)	
	Extremely severe	0 (0)	0 (0)	0 (0)	
	Mean (SD)	9.53 (5.64)	8.73 (5.55)	9.92 (5.65)	0.035

**Table 3:** Factors associated with perceived symptoms of depression, anxiety, and stress among university staff.

Factors	Symptoms of Depression		Symptoms of Anxiety		Symptoms of Stress	
	Bivariate Model cOR (95% CI)	Multivariate Model aOR (95% CI)	Bivariate Model cOR (95% CI)	Multivariate Model aOR (95% CI)	Bivariate Model cOR (95% CI)	Multivariate Model aOR (95% CI)
Gender						
Men	1.00	1.00	1.00		1.00	
Women	1.76 (1.12, 2.79) *	1.62 (1.01, 2.65)	1.13 (0.77, 1.68)		1.55 (0.86, 2.78)	
Age groups (years)						
<40	1.62 (1.07, 2.44) *	1.58 (1.02, 2.45) *	1.05 (0.72, 1.53)		1.12 (0.69, 1.97)	
≥40	1.00	1.00	1.00		1.00	
Marital status						
Single	1.47 (0.84, 2.57)		1.07 (0.63, 1.82)		1.00	
Married	1.00		1.00		1.08 (0.52, 2.23)	
Household income classification						
B40	2.44 (1.31, 4.55) *		1.67 (0.98, 2.86)		1.07 (0.50, 2.28)	
M40	1.75 (0.97, 3.13)		1.50 (0.92, 2.44)		0.88 (0.43, 1.77)	
T20	1.00		1.00		1.00	
Education level						
Secondary or less	1.21 (0.76, 1.93)		1.18 (0.77, 1.82)		1.69 (0.85, 3.34)	2.20 (0.90, 5.42)
Tertiary	1.00		1.00		1.00	1.00
Occupation categories						
Academic	1.53 (0.91, 2.55)	1.61 (0.91, 2.83)	1.37 (0.87, 2.17)		1.64 (0.89, 3.02)	
Non-academic professional	2.15 (1.21, 3.81) *	2.04 (1.12, 3.72) *	1.04 (0.65, 1.67)		1.31 (0.68, 2.54)	
Non-academic support	1.00	1.00	1.00		1.00	
Duration of services (years)						
≤15	1.55 (1.03, 2.33) *		1.20 (0.83, 1.73)		1.00	
>15	1.00		1.00		1.38 (0.82, 2.32)	
Smoking status						
No	1.00		1.00		1.00	1.00
Yes	2.00 (0.99, 4.03)		1.19 (0.68, 2.08)		2.57 (0.89, 7.44)	2.56 (0.88, 7.40)
Physical activity categories						
Active PA	1.00		1.00		1.00	
Inactive PA	1.15 (0.72, 1.82)		1.15 (0.76, 1.72)		1.14 (0.64, 2.01)	
Medical conditions that required hospitalizations						
No	1.00		1.00	1.00	1.00	
Yes	1.24 (0.62, 2.46)		2.39 (1.26, 4.52) *	2.38 (1.24, 4.50) *	1.17 (0.52, 2.62)	

* Statistical significance (*p* < 0.05); cOR (crude Odds Ratio); aOR (adjusted Odds Ratio).

## Data Availability

The data presented in this study are available within the article.
